# Intermediate stages of electrochemical oxidation of single-crystalline platinum revealed by *in situ* Raman spectroscopy

**DOI:** 10.1038/ncomms12440

**Published:** 2016-08-12

**Authors:** Yi-Fan Huang, Patricia J. Kooyman, Marc T. M. Koper

**Affiliations:** 1 Leiden Institute of Chemistry, Leiden University, Einsteinweg 55, PO Box 9502, 2300 RA Leiden, The Netherlands; 2 ChemE, Faculty of Applied Sciences, Delft University of Technology, Julianalaan 136, 2628 BL Delft, The Netherlands; 3 Department of Chemical Engineering, University of Cape Town, Private Bag X3, Rondebosch 7701, South Africa

## Abstract

Understanding the atomistic details of how platinum surfaces are oxidized under electrochemical conditions is of importance for many electrochemical devices such as fuel cells and electrolysers. Here we use *in situ* shell-isolated nanoparticle-enhanced Raman spectroscopy to identify the intermediate stages of the electrochemical oxidation of Pt(111) and Pt(100) single crystals in perchloric acid. Density functional theory calculations were carried out to assist in assigning the experimental Raman bands by simulating the vibrational frequencies of possible intermediates and products. The perchlorate anion is suggested to interact with hydroxyl phase formed on the surface. Peroxo-like and superoxo-like two-dimensional (2D) surface oxides and amorphous 3D α-PtO_2_ are sequentially formed during the anodic polarization. Our measurements elucidate the process of the electrochemical oxidation of platinum single crystals by providing evidence for the structure-sensitive formation of a 2D platinum-(su)peroxide phase. These results may contribute towards a fundamental understanding of the mechanism of degradation of platinum electrocatalysts.

## Abstract (web summary)

Understanding degradation of platinum catalysts during oxygen reduction is vital for improving proton-exchange membrane fuel cells. Here, the authors identify intermediate stages in the oxidation of Pt(111) and Pt(100) in perchloric acid using *in situ* shell-isolated nanoparticle-enhanced Raman spectroscopy.

## Graphical abstract

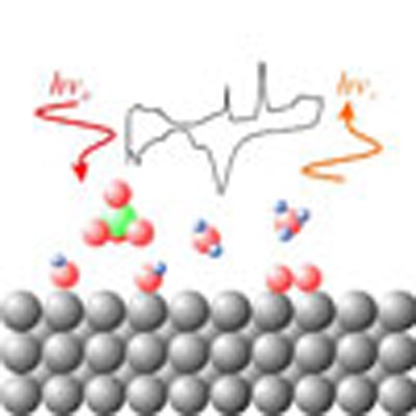

Platinum is one of the most fundamentally significant catalysts due to its widespread applications in heterogeneous catalysis and electrochemistry. The proton-exchange membrane fuel cell (PEMFC) is one of the most promising applications of Pt-based catalysts, offering a solution to the urgent energy problem as a stationary or automotive power source^1^,^2^. A PEMFC is usually composed of a Pt anode for the oxidation of fuel and a Pt-based cathode for the reduction of oxygen gas. The performance of PEMFC primarily relies on the electro-catalytic activity for the oxygen reduction reaction (ORR) of Pt catalysts^2^. Unfortunately, it has been found that the ORR activity decreases during the long-time running of PEMFC. Thus, many efforts have been devoted towards understanding the degradation of Pt catalysts during ORR^3^. Much of our understanding of the surface oxidation of platinum electrodes comes from detailed electrochemical experiments as summarized and exemplified in the works of Conway and colleagues^4^,^5^. More recently, the deployment of surface-sensitive techniques and well-defined surfaces has led to a more chemically and structurally detailed understanding of platinum surface oxidation. Cyclic voltammetry, *ex situ* X-ray photoemission spectroscopy characterization and *in situ* electrochemical scanning tunnelling microscopy studies of Pt(111) single crystals have identified majority species on the surface, such as OH_ads_ and O_ads_, and have shown that the well-defined terrace is damaged as steps and defects form at the onset of the formation of a three-dimensional (3D) oxide film, also known as the place exchange process^6^,^7^. *In situ* X-ray diffraction and energy dispersive X-ray absorption spectroscopy of supported Pt nanoparticles have illustrated this formation of a surface Pt oxide and its subsequent growth into a bulk oxide^8^. From a more practical point of view, *ex situ* electron microscopic observations have indicated that the Pt nanoparticles grow during operation and inductively coupled plasma mass spectroscopy coupled with voltammetry has shown that Pt dissolves into the electrolyte during the potential sweeping^9^,^10^,^11^. In spite of these significant advances, the nature of the surface species formed during the surface oxidation of Pt, and their dependence on surface structure, has remained ambiguous.

In this paper, we will explicitly identify the surface species formed during electrochemical oxidation of atomically flat Pt(111) and Pt(100) single crystals by *in situ* Raman spectroscopy. The recently developed method of shell-isolated nanoparticle-enhanced Raman spectroscopy (SHINERS) allows obtaining Raman spectra of surface species on single crystals covered by chemically inert silica-coated Au nanoparticles (Au@SiO_2_)^12^,^13^. Up to now, SHINERS has been successfully used to study the adsorption behaviour of small molecules on Au or Pt single crystals, such as pyridine, CO oxidation, room temperature ionic liquids and so on^13^,^14^,^15^,^16^,^17^. SHINERS also has been used to examine the surface composition of Ni-based alloys produced by electrochemical oxidation^18^. Previously, electrochemical surface-enhanced Raman spectroscopy has been used to reveal reaction intermediates on nano-structured surfaces^19^,^20^,^21^,^22^,^23^,^24^,^25^,^26^. The attempt in the present work to characterize electrochemical reaction intermediates on well-defined single crystals extends the application of Raman spectroscopy to the initial stages of surface oxidation of atomically defined catalytically relevant electrochemical interfaces. Our results will give new insights into the structure-sensitive surface oxidation of platinum single crystals in acid media, and provide evidence for a two-dimensional (2D) platinum surface (su)peroxide phase formed on Pt(111) before 3D platinum-oxide formation.

## Results

### Voltammetry and SHINERS of Pt(111) and Pt(100)

**Figure 1:**
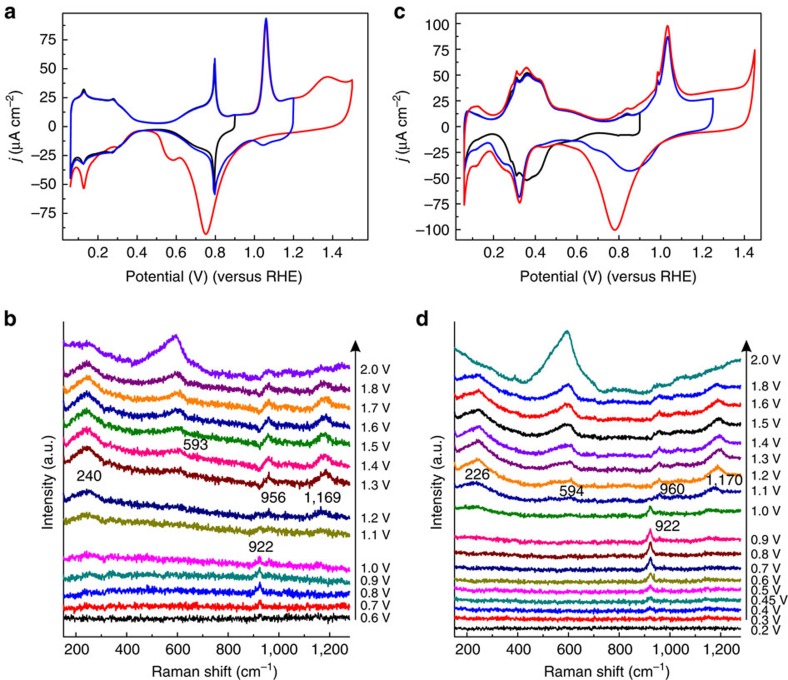
Cyclic voltammograms and potential dependent Raman spectra of Pt(111) and Pt(100). (**a**) Voltammogram of Pt(111) in 0.1 M HClO_4_ electrolyte; scan rate 50 mV s^−1^. (**b**) SHINERS spectra of Pt(111) at the indicated potentials. (**c**) Voltammogram of Pt(100) in 0.1 M HClO_4_ electrolyte; scan rate 50 mV s^−1^. (**d**) SHINERS spectra of Pt(100) at the indicated potentials. The SHINERS spectra were recorded with the potential stepped positively; every spectrum was collected in 55 s.

Figure 1a shows a cyclic voltammogram of Pt(111) in 0.1 M HClO_4_ electrolyte. It exhibits the characteristic reversible peaks in the potential regions of 0–0.4 V and 0.6–0.9 V versus reversible hydrogen electrode as a reference (RHE). If the potential is scanned to more positive values, the voltammogram is no longer reversible. A sharp peak at 1.1 V is followed by a broad peak at 1.3 V in the forward scan, whereas a broad cathodic peak is observed at ca. 0.7 V in the back scan. The reversible plateau peak at the potentials of 0–0.4 V has been ascribed to the adsorption of hydrogen atoms (H_ads_) on the surface^27^,^28^. Previous spectroscopic evidence for the formation of H_ads_ in this potential window comes from different infrared reflection techniques using Pt(111) or polycrystalline Pt (refs 29, 30, 31). The Raman spectra on Pt(111) to be described below were recorded at potentials ranging from 0.6 to 2.0 V, to reveal the surface species involved in the oxidation. The blank voltammetry of Pt(111) in the presence of the Au@SiO_2_ nanoparticles, as shown in Supplementary Fig. 1, shows that the main voltammetric features of Pt(111) are retained and that the nanoparticles block part of the surface but otherwise do not appear to influence significantly its electrochemical properties.

The development of the different spectral features on Pt(111), as shown in Fig. 1b, corresponds well to the appearance of the various peaks in the voltammogram. As the potential increases from 0.6 to 1.0 V, a characteristic band at 922 cm^−1^ is the only significant band in the spectra. At a potential of 1.1 V, bands at 240, 956 and 1,169 cm^−1^ appear, with the band at 922 cm^−1^ disappearing rapidly. These bands must reflect the chemical nature of the surface phase formed in the voltammetric peak at 1.05–1.15 V. At potentials above 1.3 V, a new broad band centred at ca. 593 cm^−1^ appears, with the other three features remaining visible. For the surface oxidation of the Pt(100) electrode, a similar correspondence between cyclic voltammogram and Raman spectral features is observed, as shown in Fig. 1c,d. However, there are two important differences with Pt(111): (i) on Pt(100), the band at 922 cm^−1^ is observed at potentials as low as 0.4 V; and (ii) the band at 594 cm^−1^ appears simultaneously with the other three bands (226, 960 and 1,170 cm^−1^) at 1.1 V.

### Hydroxyl phase on Pt(111)

**Figure 2:**
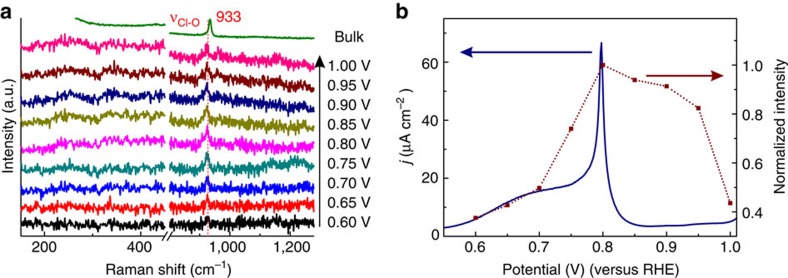
Raman spectra of the hydroxyl phase on Pt(111). (**a**) Potential dependent Raman spectra and (**b**) the Raman intensity of the band at 922 cm^−1^ compared with the electrochemical current as obtained by scanning with 50 mV s^−1^ on a Pt(111) electrode in 0.1 M HClO_4_ electrolyte. The blue line in **b** is the voltammetric current and the squares correspond to the Raman intensities estimated from **a**. The Raman spectra shown in **a** were collected during 55 s.

In light of the correspondence between the cyclic voltammogram and the Raman spectral features, these Raman spectra should give us information about the dominant surface species at each potential. Figure 2a represents potential-dependent Raman spectra of Pt(111) at the potentials from 0.6 to 1.0 V. The dominant band at 922 cm^−1^ is very close to that of the symmetric stretching mode (υ_Cl-O_, A_1_) of ClO_4_^−^ in the electrolyte. We assign this band to ClO_4_^−^ very near the Pt(111) surface. The observed Stark effect^32^,^33^,^34^,^35^,^36^ suggests that this ClO_4_^−^ is located in the compact part of the interfacial double layer; however, we do not think that it is specifically adsorbed, as explained below. The potential dependence of the intensity of this feature is plotted in Fig. 2b, showing a maximum intensity in correspondence with the maximum in the current near 0.8 V. According to the pH-dependent cyclic voltammograms in electrolytes containing ClO_4_^−^, F^−^ and OH^−^, and according to X-ray photoelectron spectroscopy, it has been proposed that the reversible peak in this potential window is due to the reversible formation of OH_ads_ species on the Pt(111) surface^37^,^38^:


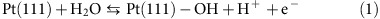


The Raman spectra in Fig. 1 do not appear to give any explicit spectroscopic evidence for the formation of OH_ads_ itself, which reaches a maximum coverage estimated to be ca. 1/3 (ref. 39). The intensity of surface enhanced Raman scattering relies on many factors, not only on the amount of species but also on the Raman scattering cross-section and the local enhancement. The intensity of the perchlorate band is very weak (several counts per second), which is quite close to the limitation of our charge-coupled device detector, meaning that the Raman scattering cross-section of Pt-OH must be even smaller than that of ClO_4_^−^. However, we emphasize that the qualitative potential dependence of the perchlorate band, as shown in Fig. 2b, was reproducible. Note that, with the exception of the data point at 1.0 V, the Raman intensity of the perchlorate signal qualitatively follows the integral of the voltammetric peak. The data point at 1.0 V lies at the onset of the second oxidation peak, for which the perchlorate signal decreases to zero intensity (Fig. 1b), and since the time of the collection of the Raman spectra is ca. 50 times longer than that of the voltammogram shown in Fig. 2b, this data point is likely to be influenced by the state of the surface generated in the voltammetric peak at 1.05 V. We therefore propose that the formation of OH on Pt(111) is associated with a specific interaction of ClO_4_^−^ with the OH_ads_ layer, and it is this ClO_4_^−^ that is observed in the spectra:


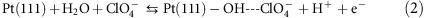


This interpretation relates to recent results of Attard *et al.*^40^ on the specific adsorption of perchlorate on Pt single-crystal electrodes, which they concluded from their observation that the cyclic voltammetry is dependent on the perchloric acid concentration. Our conclusion is different from Attard's, as we suggest that the perchlorate interacts with the OH adlayer, instead of specifically adsorbing onto the Pt(111) surface. We note that the same voltammetric curve as in Fig. 1a has been observed in acidic hydrofluoric acid (HF) and methanesulfonic acid^6^,^41^, both also considered to be non-specifically adsorbing electrolytes. Also, various density functional theory (DFT) calculations predict OH_ads_ formation in exactly the observed potential window^42^. Therefore, we consider the current flowing in the ‘butterfly' region between 0.55 and 0.85 V as being due to the formation of adsorbed OH; however, the exact shape of the butterfly is sensitive to the interaction with perchlorate. As a result, we propose that the observation of the perchlorate band in the Raman spectra is an indirect indicator of the formation of the OH adlayer. Our proposition is in agreement with the conventional wisdom that perchlorate is not specifically adsorbed, but nevertheless allows for a (small) effect of perchlorate on the voltammetric response of OH adsorption. However, the details of the interaction between ClO_4_^−^ and Pt(111)–OH are not fully understood and could well be more nonlinear than suggested by Fig. 2b, and may involve the competitive aspects referred to by Attard *et al.*^40^ We will still have more to say about the interaction between perchlorate and OH_ads_ when we will discuss the Pt(100) results in more detail on the basis of the understanding of the other bands in the Raman spectra.

### Surface (su)peroxide phase on Pt(111)

**Figure 3:**
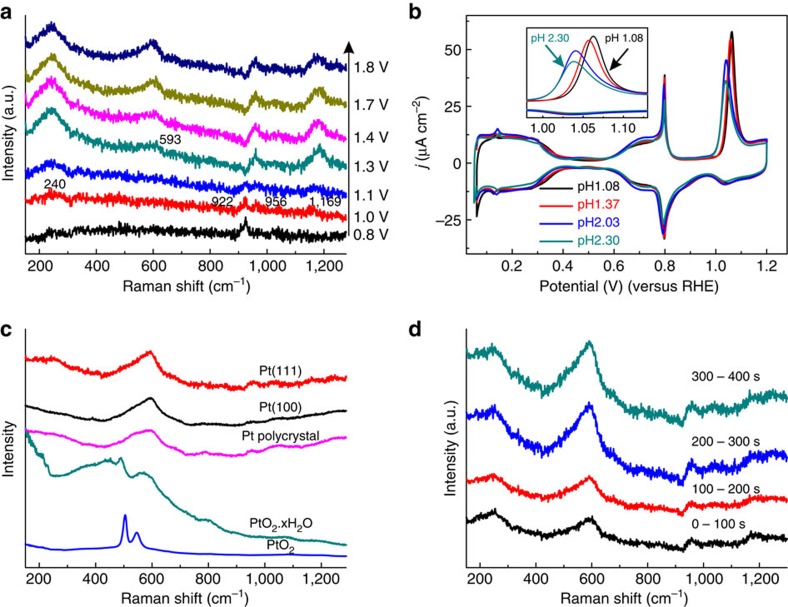
Raman spectra of (su-)peroxide and oxide formation. (**a**) Potential dependent Raman spectra of Pt(111) in 0.1 M HClO_4_ electrolyte; (**b**) pH-dependent cyclic voltammograms of Pt(111) in HClO_4_/NaClO_4_ electrolyte with a constant concentration of 0.1 M ClO_4_^−^ with a scan rate of 50 mV s^−1^; (**c**) Raman spectra of Pt(111), Pt(100) and polycrystalline Pt at 2.0 V, and normal Raman spectra of amorphous PtO_2_·xH_2_O and α-PtO_2_; (**d**) oxidation time-dependent Raman spectra of Pt(111) at 2.0 V.

As illustrated in Fig. 3a, at a potential above 1.0 V, the band at 922 cm^−1^ is rapidly replaced by the features at 240, 956 and 1,169 cm^−1^, indicating that ClO_4_^−^ does not interact strongly with the new surface species that is formed at this potential. To the best of our knowledge, this is the first observation of these bands on Pt. The appearance of these features coincides with the observation of the peak at 1.1 V in the voltammogram. The recent publication by Attard *et al.*^40^ showed that this peak shifts to higher potential with a higher concentration of HClO_4_ in solution, which was attributed to the competition between ClO_4_^−^ adsorption and water dissociation on the Pt(111) surface. The Raman results indeed suggest that perchlorate leaves the interface in this potential window. However, the pH value of the electrolyte is also dependent on the concentration of HClO_4_ used in the experiment. To specifically probe the role of the pH, we carried out pH-dependent measurements with a constant concentration of 0.1 M ClO_4_^−^, as shown in Fig. 3b. This experiment shows a small but significant shift of the peak potential from 1.062 to 1.038 V (on the reversible hydrogen electrode (RHE) scale) in response to the variation of pH from 1.08 to 2.30. Although changes in pH inevitably involve changes in the Na^+^ concentration, cation effects on the OH adsorption peaks have only been found in alkaline media and not in acidic perchloric acid, so that we exclude substantial effects of cations on the observations in Fig. 3b (ref. 43). The peak around 1.05 V and its corresponding charge are usually explained by the following surface reaction^7^


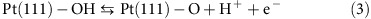


However, such a reaction cannot easily explain the observed pH shift of the peak potential on the RHE scale.

According to electron energy loss spectroscopy data of oxygen on Pt(111) in ultra high vacuum, a vibrational spectrum of atomic oxygen on Pt(111) shows a single vibration frequency of Pt-O at ca. 490 cm^−1^, whereas adsorbed molecular oxygen on Pt(111) exhibits three vibrational features at 390, 710 and 870 cm^−1^ (refs 44, 45). The vibrational features observed in our experiments at 240, 956 and 1,169 cm^−1^ are not straightforwardly explained by either of these adsorbates. By comparing spectra of platinum-dioxygen complexes (see ref. 46 and references therein), we note that peroxidic O–O stretches typically occur in the 800–900 cm^−1^ range, and that superoxidic O–O has a vibrational frequency of ca. 1,150 cm^−1^. Accordingly, Gland *et al.* assigned the electron energy loss spectroscopy band at 870 cm^−1^ to O_2_ on Pt(111), and attribute the bands at 390 and 710 cm^−1^ to the Pt-O stretch of the adsorbed peroxo, and to oxygen adsorbed on defects, respectively^44^,^45^. DFT calculations of O_2_ on Pt(111) have shown that the O–O stretching frequency is in fact very sensitive to the (local) electric field, and may vary from 750 to 1,100 cm^−1^ (ref. 47).

We have computed the vibrational frequencies of O_2_ species on Pt(111), in the absence and presence of OH on the surface, for neutral and positively charged surfaces. We used a slab model with five layers of Pt(111) surface computed with the Vienna *ab initio* simulation package code using the DFT-generalized-gradient approximation approximation; details of the geometry optimization and the vibrational frequency calculation are given in Supplementary Fig. 2 and Supplementary Table 1. These DFT calculations of the surface geometries with adsorbed O–O can reproduce the characteristic vibration frequencies at ca. 250, 970 and 1,200 cm^−1^ (Supplementary Table 1 in Supplementary Information). The 250 cm^−1^ band corresponds to the stretching mode of Pt-O_2_ vibration, whereas the bands at 970 and 1,200 cm^−1^ correspond to two different O–O stretching modes. As mentioned, the observed stretching modes of O–O (υ_OO_) are quite similar to those of peroxo and superoxo species. The calculated bond lengths of O–O are also similar with those of free O_2_^2−^ and O_2_^−^. On the basis of the combined experimental and computational data, we propose to assign the bands at 240, 956 and 1,169 cm^−1^ to platinum-peroxo and platinum-superoxo-like 2D surface oxides, respectively, where the coexistence of peroxo and superoxo species on the surface suggests that the local electric field on the surface is inhomogeneous. We emphasize that this platinum-peroxo or -superoxo surface species is different from a peroxide species generated from H_2_O_2_ interacting with Pt(111), which is in fact oxidized to O_2_ in the corresponding potential window^48^. The platinum-peroxo or -superoxo species can only be formed from the oxidation of water. We note that similar surface (su)peroxo bands have been observed recently in the electrochemical oxidation of gold and nickel-oxyhydroxide electrodes^49^,^50^. The formation of (su-)peroxo-like 2D surface oxide can be formally written as


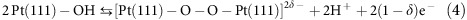


The negative charge ‘stored' in the surface (super)oxide means that reaction 4 is not a simple proton-coupled electron transfer such as reactions 1–3. Because of the negatively charged oxide, its formation is favoured at more negative ‘real' potentials, and this may explain why the peak shifts to lower potentials on the RHE scale with increasing pH, in contrast to the other peaks, which are constant on the RHE scale^51^. A simple mathematical derivation for the different pH dependence of reactions 3 and 4 illustrating this important point is given in the Supplementary Note 1. In principle, the pH-dependent interaction of perchlorate with this (su)peroxide layer may also explain the pH dependence of the peak, but note that the Raman spectra in Fig. 1 show no intensity for the perchlorate peak above ca. 1.0 V, making such an explanation less likely. A second important comment about reaction 4 concerns its bimolecular character, which has recently been postulated by both Gomez-Marin and Feliu^7^ and by Jinnouchi *et al.*^52^ to be at the origin of the irreversibility of the anodic peak at 1.05 V. They suggested the following reaction:


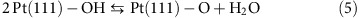


However, this reaction cannot explain the pH dependence of the voltammetric peak shown in Fig. 3b, and it cannot explain the observed Raman spectra. Finally, we note that the potential dependence of the spectral features ascribed to perchlorate and surface (su)peroxide shown in Figs 2 and 3 is reversible, that is, they disappear again when the potential is stepped back from high to low (as shown in Supplementary Fig. 3). This makes it highly unlikely that these spectral features are due to some kind of uncontrolled contamination on the surface.

### Platinum-oxide formation

At a potential of 1.3 V, a band at 593 cm^−1^ starts appearing and at 2.0 V the spectra are dominated by a broad band at ca. 600 cm^−1^, which has been assigned to the PtO_*x*_ species^53^. Figure 3c compares the spectra of Pt(111), Pt(100) and polycrystalline Pt at 2.0 V, all of which are similar with the Raman spectrum of amorphous PtO_2_·xH_2_O. (See Supplementary Fig. 4 for the spectrum in deuterated water and for a further discussion of this assignment.) The independence of spectral features on the surface orientation suggests that the surface oxide has been converted into an amorphous 3D hydrous oxide. Figure 3d shows the time dependence of the spectra at 2.0 V, where the continuously increasing intensity confirms a 3D growth of PtO_*x*_ at 2.0 V. Figure 3c also shows the Raman spectrum of α-PtO_2_ for comparison. The β-PtO_2_ has a Raman spectrum that is very different from the spectrum of α-PtO_2_ and from the hydrous PtO_*x*_ generated electrochemically; most importantly, β-PtO_2_ does not show any bands in the 500–590 cm^−1^ region, whereas α-PtO_2_ does^54^. Therefore, the PtO_*x*_ generated electrochemically appears to be mainly of α-PtO_2_ character, at least under the conditions of our experiment. The 593 cm^−1^ band does not shift significantly with potential (Fig. 1) or thickness (Fig. 3d), suggesting that this is indeed a vibrational feature from within a 3D oxide. In addition, note that the bands of the 2D surface oxide remain visible during the growth of 3D PtO_2_. This may indicate that the oxide growth is not homogeneous on the surface, as also suggested by the generation of more and more defects on a perfect single crystal after multiple voltammetric scans^7^. The observation may also relate to a conclusion drawn by Tremiliosi-Filho *et al.*^4^, who stated that the 3D oxide film must be porous so that the 2D film can be formed separately and simultaneously at the inner interface of the oxide film with the metal. The observation that the features corresponding to the 2D platinum-(su)peroxide film on Pt(111) do not change intensity for potentials higher than 1.2 V (Fig. 1b) also suggests that this film forms separately from the growing 3D α-PtO_2_ film.

### SHINERS spectra of Pt(100)

Finally, let us come back to the potential-dependent Raman spectra for the Pt(100) electrode in comparison with Pt(111) in Fig. 1d. The appearance of the perchlorate feature at 922 cm^−1^ starts at 0.4 V. This corresponds to the potential of the peak in Fig. 1c. This peak has been argued to be related to the conversion of the H-terminated Pt(100) surface to a HO-terminated Pt(100) surface^55^,^56^. This is consistent with our conclusion that perchlorate interacts with an OH-covered surface (*vide supra*), and that therefore the 922 cm^−1^ band is an indirect indicator of a hydroxylated surface. The stronger bond of OH to these surfaces as compared with Pt(111), in combination with the stabilization of OH provided by water, prevents the recombination of H and OH into water in this potential window^56^. DFT calculations also show that on more open surfaces such as Pt(100), the dissociation of water into adsorbed H and adsorbed OH is (close to) exothermic, even in the absence of water^42^,^57^. At ca. 1.0 V, four peaks start to grow simultaneously in the spectra, corresponding to same frequencies as for the 2D and 3D oxide phases formed on Pt(111). This illustrates that the 2D phase is formed separately only on Pt(111), in the voltammetric peak at 1.1 V, but that this phase is not formed separately on the other Pt facets (the Raman spectra for polycrystalline Pt are qualitatively similar to those for Pt(100)). This conclusion is also consistent with the observed peaks in the blank voltammetry in Fig. 1c, where we ascribe the voltammetric peak between 0.3 and 0.4 V to the conversion of a H-terminated to a OH-covered surface, and the peak at 1.1 V to the conversion of a OH-covered surface to a Pt(100) surface covered by a 3D oxide film.

In summary, SHINERS has been used to reveal the surface species formed in the electrochemical surface oxidation process of well-defined Pt(111) and Pt(100) surfaces. The spectra suggest that the perchlorate anion interacts with the hydroxyl phase on both Pt(111) and Pt(100). On Pt(111), the hydroxyl phase is converted in relatively sharp voltammetric peak into what we identify as a (su)peroxo-like 2D platinum surface oxide. At potentials above 1.3 V, this 2D surface oxide is followed by the formation of an amorphous 3D α-PtO_2_. On Pt(100), the spectral features are similar to those on Pt(111), but with the important difference that the 2D (su)peroxo-like surface oxide and 3D α-PtO_2_ form at the same potential. These observations elucidate the structural dependence of the surface species, as well as their identity in the electrochemical oxidation process of Pt, based on direct *in situ* vibrational spectroscopy, a result which is of potential relevance to the understanding of the degradation of the Pt catalyst during the oxygen reduction reaction. Furthermore, we have shown that *in situ* SHINERS can be a powerful tool for the identification of the intermediates on well-defined single-crystal surfaces, which can provide a better molecular understanding of electrochemical surface processes.

## Methods

### Synthesis and chemicals

SHINERS principally relies on the optical field enhanced by the surface plasmon resonance of a Au nanoparticle coated by thin SiO_2_ layer (Au@SiO_2_ NPs). The synthesis of Au@SiO_2_ NPs followed the procedure in the literature^58^. First, 2 ml 1 wt% sodium citrate solution was added into 200 ml 0.01 wt% HAuCl_4_ solution (Sigma-Aldrich). The mixture was refluxed for 1 h under vigorous stirring. After the solution was cooled down, 400 ml 1 mM ATPMS (3-aminopropyltriethoxysilane, Sigma-Aldrich) solution was added to 30 ml prepared Au solution, which was stirred for 15 min at room temperature. Then, 3.2 ml 0.54 wt% sodium silicate solution (Sigma-Aldrich) with a pH between 10 and 11 was added to the solution. To avoid contamination, the pH value was adjusted by adding HClO_4_ solution, as also used in the electrochemistry and Raman measurements. Finally, the solution was transferred into a boiling water bath and kept there for 30 min. (See Supplementary Fig. 5 for high-resolution transmission electron microscopy characterization of the prepared Au@SiO_2_ NPs). The synthesized Au@SiO_2_ NPs were spread on Pt(111) and Pt(100) crystals for Raman measurement. The solution of Au@SiO_2_ NPs was dropped onto the crystal and dried in a desiccator. Before the Raman measurement, the electrodes covered by Au@SiO_2_ NPs were applied a potential of −2.0 V in 0.1 M HClO_4_ electrolyte for cleaning, where the coverage of the particles were estimated to be <30% (ref. 59). All the Raman measurements were performed in a spectroelectrochemical cell strictly isolated from air with Ar bubbling to exclude the influence from oxygen as much as possible. The water in all experiments is produced by a Milli-Q gradient A10 system.

### Electrochemistry

The single-crystal electrodes were annealed in a methane flame and cooled down in Ar:H_2_(3:1) atmosphere. The 0.1 M HClO_4_ electrolyte was prepared from HClO_4_ (Merck, 70%). In all experiments, a reversible hydrogen electrode was used as reference, to avoid any contamination from anions in the system.

### *In-situ* Raman measurements

Raman spectra were recorded with a HR-800 (Jobin Yvon-Horiba, France) spectrometer integrated with a confocal microscope. The spectra were obtained by excitation with a He–Ne laser with a wavelength of 632.8 nm. To filter the background, the potential dependent Raman spectra were subtracted by the spectrum at a reference potential. (Pt(111) at 0.5 V and Pt(100) at 0.15 V).

### Computational details

The geometry optimization and vibrational frequency calculation of surface oxides/hydroxide on Pt(111) were performed by slab calculations with DFT using Vienna *ab initio* simulation package^60^,^61^,^62^,^63^. The projector augmented wave method^64^,^65^ and Perdew–Burke–Ernzerhof generalized-gradient approximation^66^ functional were used. An energy cutoff of 550 eV, a Monkhorst–Pack *k*-point sampling of 9 × 9 × 1 (ref. 67) and a first-order Methfessel–Paxton smearing with a sigma of 0.2 were applied^68^. The charged cell with a vacuum layer of 30 Å was used to describe the species on the surface. The Pt(111) surface is a 2 × 2 surface unit cell with the top three layers allowed to relax and two layers fixed at the bottom and a lattice constant of 3.9864 Å (ref. 57).

## Additional information

**How to cite this article:** Huang, Y.-F. *et al.* Intermediate stages of electrochemical oxidation of single-crystalline platinum revealed by *in situ* Raman spectroscopy. *Nat. Commun.* 7:12440 doi: 10.1038/ncomms12440 (2016).

## Supplementary Material

Supplementary Information Supplementary Figures 1-5, Supplementary Table 1, Supplementary Note 1 and Supplementary References.
