# Supplementary material for: Intermediate stages of electrochemical oxidation of single-crystalline platinum revealed by in situ Raman spectroscopy
Source: Nat Commun. 2016 Aug 12;7:12440. doi: 10.1038/ncomms12440 (PMC4990643; doi:10.1038/ncomms12440)
Supplement: Supplementary Information — Supplementary Figures 1-5, Supplementary Table 1, Supplementary Note 1 and Supplementary References. [file ncomms12440-s1.pdf]

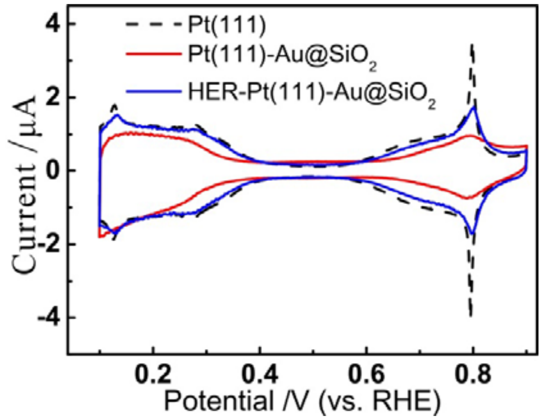

Supplementary Figure 1 Characterization of the Pt(111) covered by Au@SiO_2 nanoparticles. Cyclic voltammogram of Pt(111), of Pt(111) covered by Au@SiO_2 and of a Pt(111) electrode covered by Au@SiO_2 after treatment by the hydrogen evolution reaction, as used in SHINERS measurements in 0.1 mol/L $HClO_4$. This experiment demonstrates the cleanliness of the Pt(111) electrode covered by Au@SiO_2 as used in the SHINERS measurement, we performed a cyclic voltammetry measurement, as shown in figure S2. It can be seen that the voltammogram of hydrogen adsorption/desorption in 0.08 V ~ 0.5 V and the phase transition of OH in 0.5 V ~ 0.9 V on Pt(111) covered by Au@SiO_2 cleaned by hydrogen evolution treatment are similar with that on bare Pt(111). So the properties of Pt(111) covered by Au@SiO_2 used in the SHINERS are comparable with the conventional single crystal electrochemistry.

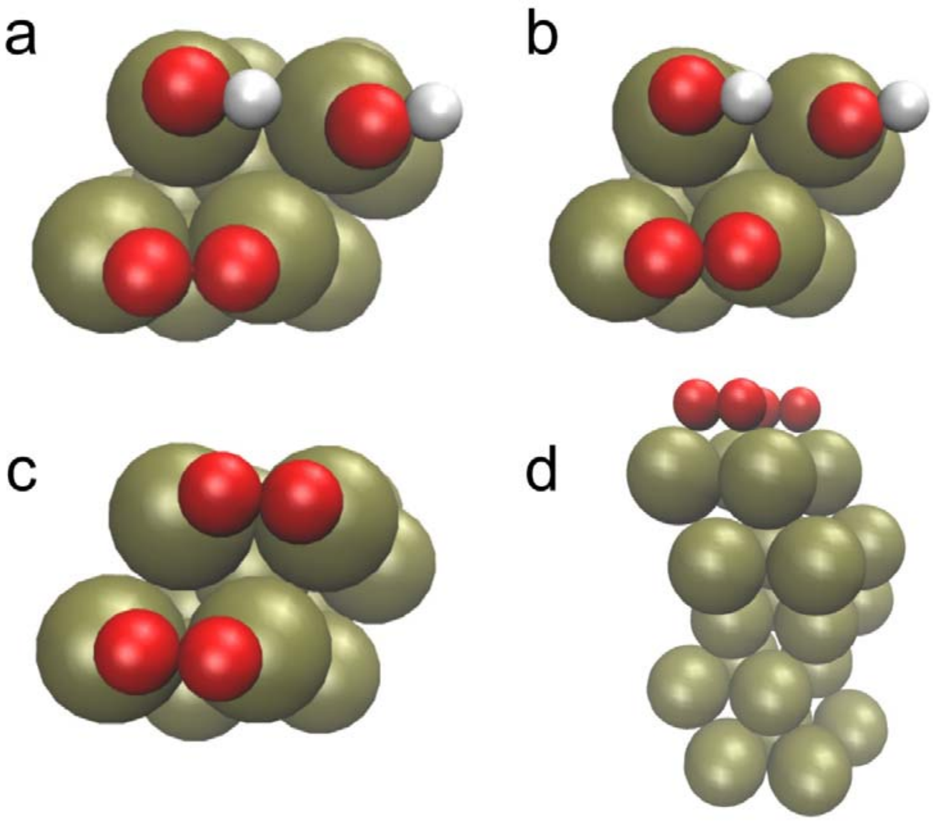

Supplementary Figure 2 Computational treatment of adsorbed O_2. Top views of the optimized super-cells of (a) Pt(111)$(OH)_2$-O_2, (b) $[Pt(111)(OH)_2\text{-}O_2]^+$, (c) $[Pt(111)\text{-}(O_2)_2]^{2+}$, and (d) the side view of $[Pt(111)\text{-}(O_2)_2]^{2+}$ (d). At an experimental electrode potential above 1.0 V, the Pt surface is covered by OH. In order to simulate the local electronic structure, some of the 1st layer Pt atoms are bonded by OH and the supercell was charged positively, as indicated in Supplementary Table 1. The optimized geometries are shown in the above figure S. Bader charge analysis program was employed to analyze the charge distribution on oxygen atoms of adsorbed O_2.[1-3]

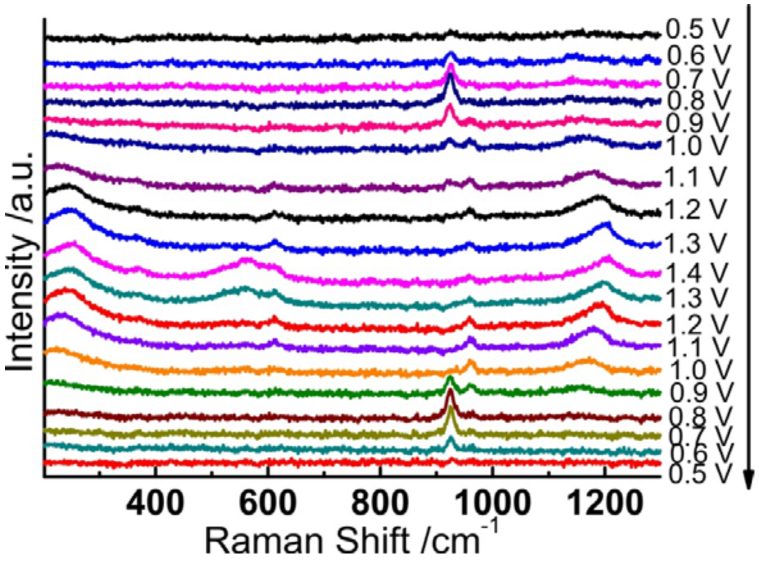

Supplementary Figure 3 Reversibility of the observed(su)peroxide features. Raman spectra of Pt(111) covered by Au@SiO_2 NPs in 0.1 M $HClO_4$ solution, with increasing and subsequently decreasing potential.

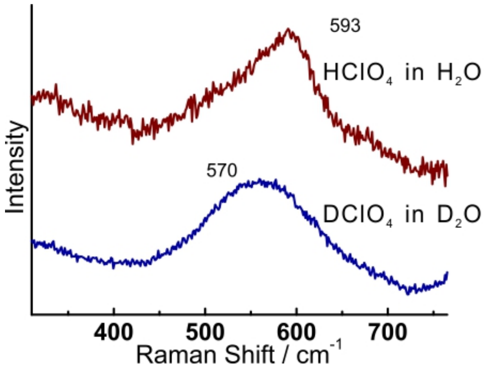

Supplementary Figure 4 Measurements in normal vs. deuterated water. Raman spectra of Pt(111) covered by Au@SiO_2 NPs at 2.0 V in 0.1 M $HClO_4$ in H_2O and 0.1 M $DClO_4$ in D_2O solutions, showing comparative Raman spectra of Pt(111) covered by Au@SiO_2 NPs at 2.0 V in 0.1 M $HClO_4$ in H_2O and 0.1 M $DClO_4$ in D_2O solutions. It can be seen that the broad peak at 593 cm^{-1} in 0.1 M $HClO_4$ in H_2O shifts to 570 cm^{-1} in 0.1 M $DClO_4$ in D_2O, which indicates the species with OH groups (including water) is involved in the formation of amorphous PtO_2•xH_2O. This observation is also similar with SERS on polycrystalline Pt film.[6] Results from EELS also suggests a $Pt(OH)_4$ structure with a similar bands in UHV.[7]

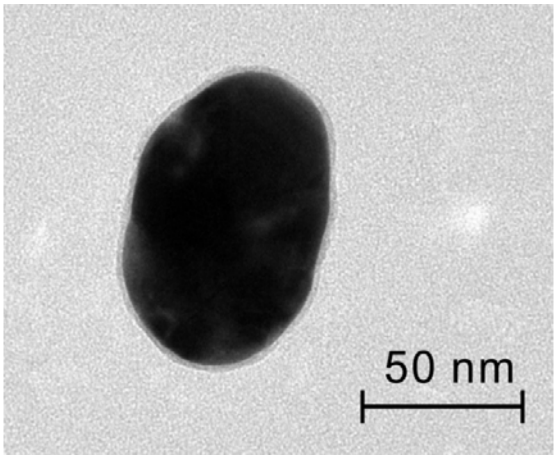

Supplementary Figure 5 Characterization of Au@SiO_2 NPs. HRTEM image of Au@SiO_2 nanoparticle (FEI TecnaiF20, FEG 200 kV) of the Au@SiO_2 nanoparticles prepared according to the procedure ascribed in the main paper.

Supplementary Table 1 Vibrational analysis and Bader charge distribution of oxygen molecules. This table lists the vibrational frequencies and bond lengths of the charged O_2 species bonded to the surfaces, in the absence and presence of OH, with different positive charges. The surface-bonded O_2 species shows similar vibrational frequencies with our Raman spectroscopic observations, and the bond lengths agree with free peroxo and superoxo, which are also consistent with our previous calculation.[4] We emphasize that these calculations are not meant to simulate closely or realistically the real system, but only to illustrate that O_2 bound to Pt(111) under suitable conditions can indeed exhibit the experimentally observed vibrational properties.

Species	Bond Length /Å	$\upsilon_{Pt\text{-}O2}$ / cm^{-1}	$\upsilon_{O\text{-}O}$ / cm^{-1}	Charge on oxygen atom / a.u.
$Pt(111)(OH)_2\text{-}O_2$	1.36	206	892	–0.24
$[Pt(111)(OH)_2\text{-}O_2]^+$	1.32	327	993	–0.14
$[Pt(111)\text{-}(O_2)_2]^{2+}$	1.29	239	1095	–0.05

Supplementary Note 1 pH dependence of reactions 3 and 4 in the main manuscript

Here we show that reactions 3 and 4 in the main manuscript exhibit a different pH dependence on the RHE scale, in correspondence with the claims made in the manuscript. We will assume that the peak potentials corresponding to reactions 3 and 4 scale in a one-to-one fashion with the corresponding equilibrium potentials, and hence we give expressions here for the equilibrium potentials of reactions 3 and 4.

Reaction 3 has an equilibrium potential given by:

$$\begin{aligned} E_3^{eq} &= E_3^{0,NHE} + \frac{RT}{F}\ln\left(\frac{\theta_O c_{H^+}}{\theta_{OH}}\right) \\ &= E_3^{0,NHE} + \frac{RT}{F}\ln c_{H^+} + \frac{RT}{F}\ln\left(\frac{\theta_O}{\theta_{OH}}\right) = E_3^{0,RHE} + \frac{RT}{F}\ln\left(\frac{\theta_O}{\theta_{OH}}\right) \end{aligned} \quad (1)$$

showing that the equilibrium and peak potential do not shift with pH on the RHE scale. In Equation 1, E_3^{eq} is the equilibrium potential of reaction 3, $E_3^{0,NHE}$ is the standard equilibrium potential of reaction 3 on the Normal Hydrogen Electrode (NHE) scale, $E_3^{0,RHE}$ is the standard equilibrium potential of reaction 3 on the Reversible Hydrogen Electrode (RHE) scale, θ_O is the coverage of the electrode with O, θ_{OH} is the coverage of the electrode with OH, c_{H^+} is the concentration (activity) of protons in solution, and *R*, *T,* and *F* have their usual meaning.

For reaction 4, we obtain the following expression for the equilibrium potential:

$$\begin{aligned} E_4^{eq} &= E_4^{0,NHE} + \frac{RT}{2(1-\delta')F}\ln\left(\frac{\theta_{O_2} c_{H^+}^2}{\theta_{OH}^2}\right) \\ &= E_4^{0,NHE} + \frac{RT}{(1-\delta')F}\ln c_{H^+} + \frac{RT}{2(1-\delta')F}\ln\left(\frac{\theta_{O_2}}{\theta_{OH}^2}\right) \\ &\approx E_4^{0,NHE} + \frac{RT}{F}\ln c_{H^+} + \frac{\delta' RT}{F}\ln c_{H^+} + \frac{RT}{2(1-\delta')F}\ln\left(\frac{\theta_{O_2}}{\theta_{OH}^2}\right) \\ &= E_4^{0,RHE} - \frac{\delta' RT \ln 10}{F} pH + \frac{RT}{2(1-\delta')F}\ln\left(\frac{\theta_{O_2}}{\theta_{OH}^2}\right) \end{aligned} \quad (2)$$

where the symbols have the same meaning as in Equation 1. Equation 2 shows that the equilibrium and peak potential shift to lower potentials with increasing pH, as observed experimentally. In the third equality, we have assumed that $0 < \delta' << 1$, for simplicity. We note that the δ' in the above equations is not exactly the same quantity as the partial charge number δ in Eq.4, as δ' is rather an electrosorption valence, which is the relevant thermodynamic quantity, and which includes details of the double layer structure as well as the partial charge number.[5]

Supplementary References

1 G. Henkelman, A. Arnaldsson & Jonsson, H. A fast and robust algorithm for Bader decomposition of charge density. *Comput. Mater. Sci.* **36**, 254-360, (2006).

2 Sanville, E., Kenny, S. D., Smith, R. & Henkelman, G. Improved grid-based algorithm for Bader charge allocation. *J. Comp.Chem.* **28**, 899-908, (2007).

3 Tang, W., Sanville, E. & Henkelman, G. A grid-based Bader analysis algorithm without lattice bias. *J. Phys.: Cond. Mat.* **21**, 084204, (2009).

4 Panchenko, A., Koper, M. T. M., Shubina, T. E., Mitchell, S. J. & Roduner, E. Ab Initio Calculations of Intermediates of Oxygen Reduction on Low-Index Platinum Surfaces. *J. Electrochem. Soc.* **151**, A2016-A2027, (2004).

5 Schmickler, W. & Santos, E. *Interfacial Electrochemistry*. 2 edn, (Springer-Verlag Berlin Heidelberg, 2010).

6 Zhang, Y., Gao, X. & Weaver, M. J. Nature of surface bonding on voltammetrically oxidized noble metals in aqueous media as probed by real-time surface-enhanced Raman spectroscopy. *J. Phys. Chem.* **97**, 8656-8663, (1993).

7 Peuckert, M. & Ibach, H. Vibrational spectra of an oxidized platinum electrode. *Surf. Sci.* **136**, 319-326, (1984).
